# Teacher cognition in teaching intercultural communicative competence: A qualitative study on preservice Chinese language teachers in Hong Kong SAR, China

**DOI:** 10.3389/fpsyg.2022.939516

**Published:** 2022-07-28

**Authors:** Yang Frank Gong, Chun Lai, Xuesong (Andy) Gao, Guofang Li, Yingxue Huang, Lin Lin

**Affiliations:** ^1^Faculty of Education, University of Macau, Macau, Macau SAR, China; ^2^Faculty of Education, University of Hong Kong, Hong Kong, Hong Kong SAR, China; ^3^School of Education, Faculty of Arts and Social Sciences, University of New South Wales, Sydney, NSW, Australia; ^4^Department of Language and Literacy Education, Faculty of Education, University of British Columbia, Vancouver, BC, Canada; ^5^Keystone Academy, Beijing, China; ^6^International Culture Exchange School, Shanghai University of Finance and Economics, Shanghai, China

**Keywords:** preservice teachers, teacher cognition, intercultural communicative competence, Chinese as an additional language, teacher education

## Abstract

The purpose of this study is to examine preservice Chinese language teachers’ cognition in teaching intercultural communicative competence. In the study we collected data through in-depth interviews with seven preservice teachers in a Master of Education program (Teaching Chinese as a Second Language, TCSL) at a university in Hong Kong SAR, China. The findings indicated that the participants had a relatively positive attitude and inclination toward the development of students’ intercultural communicative competence, while their conceptualizations of culture tended to be static and ambiguous. In addition, the participants’ objectives in teaching intercultural communicative competence were found to be more attitude-than knowledge- or skill-oriented. The study offers valuable insights that preservice language teachers’ cognition plays a crucial role in their future professional development and calls for curricular innovations with intercultural aims in teacher education programs.

## Introduction

Over the last 20 years the main orientation of language education has expanded from communicative competence to intercultural communicative competence, and language teachers are now expected to enable learners to become interculturally competent (Bennett, 2004; Feng et al., 2009; Tolosa et al., 2018). Intercultural communicative competence is related to the capability to communicate appropriately and effectively with people from other linguistic and cultural backgrounds (Deardorff, 2006; Byram, 2014). While there is no single definition of intercultural communicative competence, it is generally considered to include four dimensions: attitudes, knowledge, skills, and behaviors (Perry and Southwell, 2011). For example, Byram’s definition (Byram, 1997, p. 34) identifies five elements that affect intercultural communication, namely attitudes of openness and curiosity (*savoir-être*), knowledge of one’s own as well as other social groups’ cultural settings, norms, and interaction processes (*savoirs*), skills of interpreting and relating (*savoir-comprendre*), skills of discovery and interaction (*savoir-faire*), and critical cultural awareness (*savoir s’engager*). These elements are in line with analyses of linguistic, sociocultural, and discourse competence in intercultural encounters (Porto, 2019).

An intercultural orientation to language teaching and learning can help learners to foster the ability to interact successfully with others across language and cultural differences and prepare them for global citizenship. However, the development of students’ intercultural communicative competence represents a significant challenge for many language teachers in a variety of educational contexts (e.g., Baker and Fang, 2021: the United Kingdom and China; Gandana and Parr, 2013: Indonesia; Fernández-Agüero and Chancay-Cedeño, 2019: Ecuador; Poehner and Pasterick, 2021: the United States). At the same time, it also raises a new professional need to prepare preservice (student) teachers for the challenge of developing learners’ intercultural communicative competence in their future teaching (Romijn et al., 2021). In particular, preservice teachers need to develop the ability to conduct effective pedagogical design for intercultural education in language classrooms. Because teacher cognition is “expected to significantly influence the ways in which teachers interpret and engage with the problems of practice” (Skott, 2014, p. 9), investigating language teachers’ “unobservable cognitive dimension of teaching” (Borg, 2003, p. 81) has been identified as a crucial means to address this challenge. In addition, it is vital to obtain more insights into the dimensional structure of intercultural teaching cognition if we want to address specific cognitive systems as part of preservice teachers’ pedagogical development.

Understanding what teachers understand is assumed to underlie teacher education, development, and instructional behaviors. Extensive research has been conducted over the past two decades to explore the relationship between teacher cognition and instructional practices, and the influence of these factors on student learning achievement in language teaching (Borg, 2003; Burns et al., 2015; Kubanyiova and Feryok, 2015). These studies consistently report that language teachers’ cognition links their teaching practices and students’ learning experiences. However, preservice teachers’ cognition has remained under-examined to date, and relatively limited attention has been paid to their “inner lives” (Kubanyiova and Feryok, 2015, p. 436) in terms of their teaching intercultural communicative competence, although it is recognized that the preservice years are characterized by processes of confronting complicated conceptions about cultural diversity and an active construction of what it means to teach.

The majority of the existing studies have focused on teacher cognition in developing students’ intercultural communicative competence where English is taught and learnt as a second or foreign language, predominantly in Europe and the United States (Ghavamnia, 2020). Studies have also explored the intercultural teaching of language teachers teaching other Western European languages such as French (Marshall and Bokhorst-Heng, 2018), German (Feryok and Oranje, 2015), and Spanish (Belpoliti and Pérez, 2016). Most of these studies have examined language teachers’ attitudes and beliefs concerning what they think about intercultural communicative competence and how they might integrate it into their daily teaching practices. Studies have also investigated factors influencing language teachers’ cognition in teaching intercultural communicative competence, generating insights for teacher educators and trainers to enhance language teachers’ intercultural teaching.

However, few studies on the issue of language teacher cognition have explored teachers of non-English courses or in non-European language countries (Gong et al., 2018a; Ghavamnia, 2020). Specifically, little attention has been focused on teachers who help students to develop intercultural communicative competence in the context of learning Chinese as an additional language. These teachers may demonstrate different features in their teaching of intercultural education compared with their English-teaching counterparts. Hence, the present study aims to examine how preservice Chinese language teachers perceive teaching intercultural communicative competence.

## Literature review

### Language teachers’ cognition in teaching intercultural communicative competence

Teacher cognition has been defined as “what teachers know, believe, and think” (Borg, 2003, p. 81). Language teacher cognition relates to “the complex, practically oriented, personalized, and context-sensitive networks of knowledge, thoughts and beliefs that language teachers draw on in this work” (Borg, 2006, p. 272), and the last fifty years have witnessed a continuously growing interest in this issue to provide insight into their instructional behaviors and further influence students’ learning experiences (Cathcart and Olsen, 1976; Guo et al., 2019). Specifically in terms of language teachers’ intercultural teaching, the volume of research on their cognition has increased over the past 20 years. Because of the status of English as a global lingua franca and its considerable influence on the language education landscape, most studies on language teachers’ cognition in teaching intercultural communicative competence have been conducted in the context of teaching and learning English as a second/foreign language (ESL/EFL) (Liddicoat and Scarino, 2013; Gong et al., 2018a). For example, in a qualitative study Larzén-Östermark (2008) interviewed 13 Finland-Swedish teachers of English in grades 7–9, and reported that most participants tended to define culture as “factual knowledge” (p. 527) and perceived that intercultural teaching was mainly aimed at promoting students’ cultural knowledge. At the same time, few participants showed an orientation toward improving students’ intercultural attitudes, such as openness and tolerance toward other different cultures. Young and Sachdev (2011) investigated experienced teachers’ beliefs and practices related to developing students’ intercultural communicative competence in English language programs in the United Kingdom, the United States, and France and revealed that while the teachers believed in the relevance of interculturality to their teaching, they “seemed unable or unwilling to put it into practice” (p. 95). Similarly, a recent study conducted by Safa and Tofighi (2022) with 100 preservice and 100 in-service EFL teachers in Iran found that both preservice and in-service participants shared a positive attitude and inclination toward incorporating intercultural communicative competence into their classroom instructional practices. However, this study also indicated a disparity between the participants’ stated beliefs and their actual practices through classroom observations.

In summary, most scholars consider that language teachers’ cognition plays a crucial role in shaping their instructional decisions and practices in terms of developing students’ intercultural communicative competence (Sercu, 2002; Oranje and Smith, 2018). Even though there is no single agreed definition of language teacher cognition in research on teaching intercultural communicative competence, the existing studies on this issue have mostly focused on second or foreign language (SL/FL) teachers’ pedagogical knowledge about, and their objectives in, developing students’ intercultural communicative competence. On the one hand, a SL/FL teacher needs to possess adequate cultural knowledge of her/his own community and the target language community, and it is an essential precondition to be able to display, relate, and interpret the similarities and differences between cultures to students. On the other hand, a SL/FL teacher is often expected to not only improve students’ intercultural knowledge, but also develop their intercultural attitudes, skills, and behaviors to address issues in practical intercultural encounters. Current studies have unanimously indicated that most language teachers have only a vague conceptualization of intercultural communicative competence theoretically, and they take little pedagogical account of students’ holistic intercultural development (e.g., Gong et al., 2018a; Agostinetto and Bugno, 2020). Moreover, there is little empirical investigation of teacher cognition in developing students’ intercultural communicative competence in education contexts involving languages other than English, especially Chinese as an additional language.

### Chinese language teachers’ cognition in teaching intercultural communicative competence

Over the past decade Chinese has emerged as an increasingly important language that is taught and learnt as an additional language worldwide (Ma et al., 2017; Gong et al., 2018b,^2020^). Teaching intercultural communicative competence has become an integral part and a core goal of curriculum design and implementation in the teaching of Chinese as an additional language. For instance, teachers and schools have been reported to use different strategies to enhance the integration of international mindedness into the subject of Chinese language in Hong Kong SAR, China’s International Baccalaureate diploma programs (IBDP) (Lai et al., 2014). The “*Standards for Teachers of Chinese to Speakers of Other Languages*” published by Hanban (2007) (now referred to as the Center for Language Education and Cooperation), the official guidebook for Chinese as an additional language teachers, also lists knowing “culture and communication” as one of the five important modules in Chinese language teaching.

Despite this emphasis on student intercultural development in the context of teaching Chinese as an additional language, few studies have explored Chinese language teachers’ cognition in relation to developing students’ intercultural communicative competence, especially concerning preservice teachers. Previous research has mainly focused on in-service Chinese teachers’ cognition and practices in developing students’ intercultural communicative competence; for instance, Gong et al. (2018a) investigated 43 Chinese as a second language teachers’ intercultural teaching cognition and the contextual factors (e.g., academic atmosphere) that influenced it. Their findings suggested that the participants had more pedagogical cultural knowledge about Chinese people’s daily lives, traditions, folklore, and pop culture, than about their values, ethical and social groups, literature, international relations, and arts. In terms of their objectives in teaching intercultural communicative competence, the teachers highlighted the enhancement of students’ intercultural skills more their intercultural knowledge and attitudes. Meanwhile, through interviews and classroom observations with 16 Chinese language teachers from Hong Kong SAR, China’s international schools, Gong et al. (2022) found that the teachers’ different professional and sociocultural identities interacted with their intercultural teaching perceptions and practices. In particular, while broadly defined encompassing identities such as “cultural bridge” could mediate the teachers’ intercultural teaching with a primary focus on improving students’ attitudes toward different cultures, relatively narrow and ethnocentrically positioned identities such as “Chinese culture bearer” tended to shape the one-way pedagogy of literally transmitting Chinese cultural knowledge in teaching intercultural communicative competence.

Overall, although there have been some attempts to explore language teachers’ cognition in teaching intercultural communicative competence, few studies have concentrated on preservice teachers’ cognition related to promoting students’ intercultural development in language education, especially in the context of languages other than English. Additionally, it has been argued that teachers’ preservice development profoundly influences their pedagogical beliefs and practices (Friesen and Besley, 2013). More specifically, preservice teachers’ cognition regarding the nature of intercultural education is generally seen as a relevant precondition to becoming effective practitioners of teaching intercultural communicative competence (Senyshyn, 2018). Hence, the present study aims to examine the cognitions of preservice Chinese as an additional language teachers regarding teaching intercultural communicative competence. Specifically, the study addresses the following question:

Research Question: What are preservice Chinese language teachers’ cognitions related to teaching intercultural communicative competence, including their knowledge about and their objectives in teaching?

## Methodology

### Research context

This research was conducted in Hong Kong SAR, China, which had been a British colony for more than 150 years before the transfer of sovereignty from the United Kingdom to the People’s Republic of China (PRC) in 1997. This historical background has created a multilingual and multicultural *status quo* in Hong Kong SAR, China. In terms of language use, while Cantonese is the dominant daily language used by almost 90% of the population in Hong Kong SAR, China, English is also an official language widely used in government, business, education, and legal matters. Additionally, immigrants and expatriates from Western and Asian nations/regions also enhance the role of English in daily communication. Putonghua (also referred to as Mandarin outside the Chinese mainland), the national lingua franca of the PRC, has become more and more important in the past 15 years because socio-economically advantaged Hong Kong SAR, China parents expect to “develop their children as elite bilinguals able to transcend linguistic and cultural boundaries” (Davison and Lai, 2007, p. 125).

Because of the increasing influence of Putonghua in Hong Kong SAR, China, the majority of international schools have started to offer their students courses in Putonghua as an additional language or as a compulsory second language up to secondary school age (Lai et al., 2016). Specifically, the Hong Kong SAR, China Government defines international schools as schools that “follow a full non-local curriculum designed for the needs of a particular cultural or linguistic group and/or for students who do not sit for local examinations” (EDB, 2021). These international schools are primarily self-financing and usually offer non-local curricula from places such as the United Kingdom, the United States, Singapore, or Australia, as well as the International Baccalaureate (IB) curriculum. This study focuses on prospective teachers who had pursued their Master of Education degrees and internship experiences in international schools in Hong Kong SAR, China, which usually pay significant attention to developing their students’ global citizenship and intercultural development, and which require teachers to concentrate on promoting these aspects through their instructional approaches and curriculum design.

### Participants

The participants in the present research were recruited from a Master of Education program (Teaching Chinese as a Second Language, TCSL) at a university in Hong Kong SAR, China. Seven out of 24 prospective teachers in this program voluntarily took part in the research, including six females and one male. The participants were all ethnic Chinese who had received their K-12 and undergraduate education in mainland Chinese universities, and most did not speak Cantonese as their first or mother language. Table 1 summarizes the profiles of the seven participating prospective teachers. The names used are all pseudonyms.

**TABLE 1:** Study participants.

No.	Name	Gender	Native language(s)	Curriculum’s origin	Teaching experience
1	Xiao	Female	Putonghua	IB	2 years
2	Meng	Female	Putonghua	IB	No
3	Ying	Female	Minnan Dialect, Putonghua	IB	No
4	Lan	Female	Putonghua	IB	1 year
5	Zeng	Female	Cantonese, Putonghua	IB	No
6	Min	Female	Putonghua	IB	No
7	Lu	Male	Putonghua	IB	No

These prospective teachers came to Hong Kong SAR, China to study in the one-year full-time teacher education program, which is designed to prepare teachers for teaching Chinese as an additional language at schools in Hong Kong SAR, China. In the program all the prospective teachers need to complete six compulsory courses and three elective ones, such as Teaching the Chinese Language in International Contexts. At the same time the course also includes a heavy teaching practicum component, with all the prospective teachers taking part in this study being assigned to different international schools adopting the IB curriculum. Each prospective teacher worked with a school mentor from the Chinese language panel designated by each international school. The practicums lasted about 3 months, during which time the participants were involved first in in-class observations and then in daytime teaching practice at the schools.

### Data collection and analysis

The study was based on individual interviews with the participants carried out in their native language, Putonghua, so that they could express themselves more freely. Each participant took part in one face-to-face interview at the end of their teaching practicum. Overall, the interviews were aimed at eliciting the participants’ knowledge about and objectives in developing their students’ intercultural communicative competence in Chinese as an additional language classrooms. Each interview lasted about 1 h. Elaboration and clarification questions were asked during the interviews to gain in-depth understanding and confirm the interviewees’ intended meaning.

All the interviews were audio-taped, transcribed verbatim in Chinese, and double-checked for accuracy. The researchers read through the interview transcripts five times to familiarize themselves with the data, and meaningful parts of the text that struck the researchers as interesting or important to the research were highlighted and coded. Thematic analysis was adopted to analyze the interview data, carried out in deductive and inductive phases (Braun and Clarke, 2012). The data were first analyzed into predetermined themes to examine different aspects of intercultural teaching, informed by both the current literature and the data. These themes included: (1) teachers’ knowledge about developing students’ intercultural communicative competence, and (2) teachers’ objectives in developing students’ intercultural communicative competence. The interview data were then analyzed inductively to generate concrete categorizations under each predetermined theme. For instance, under the predetermined theme *teachers’ objectives in developing students’ intercultural communicative competence*, codes like “knowing about Chinese festivals or Chinese folk customs” and “understanding Chinese people’s thinking styles” were aggregated into the category *intercultural knowledge-oriented objective*. The initial codings of the overarching categories were also compared across the seven participants to identify similar or repeated responses and contrasting instances (Charmaz, 1990). Two experienced researchers were invited to conduct peer-debriefing sessions during the interview data analysis to minimize bias (Onwuegbuzie and Leech, 2007).

We were aware that the researchers’ own understandings and views can interfere with the “objectivity, reflexivity and authenticity of [the] research project” (Kanuha, 2000, p. 444), and thus we conducted participant checking after the data collection and the write-up in order to enhance the rigor of the research and the credibility of the research findings. In order to ensure the accuracy of the data and the trustworthiness of the subsequent analysis, all interview transcriptions were sent back to the participants to see whether there was anything they would like to correct, clarify, or add to inform the analysis and help us develop new ideas and interpretations (Birt et al., 2016). One participant made minor annotations, and the other six participants returned the documents without additional comment.

## Findings

Overall, the data analysis suggested that while the preservice Chinese language teachers in this study considered themselves sufficiently familiar with intercultural communicative competence, their conceptualization of culture tended to be ambiguous and static, mostly focusing on traditional cultural knowledge (e.g., customs, routines, folklore). The participants’ accounts revealed that all of them believed it was crucial to accommodate a curriculum orientation toward students’ intercultural communicative competence. In particular, most participants paid more attention to developing students’ intercultural attitudes than their intercultural knowledge and skills.

### Chinese as an additional language teachers’ knowledge about teaching intercultural communicative competence

Reflecting the assumption that “[c]ulture has always been at the centre of discussions in intercultural education” (Dervin, 2016, p. 9), the interview data revealed that all the participants (7/7) showed a conceptual understanding of interculturality, treating culture as a concept that was too broad to be precisely defined. In particular, they believed that culture embraced or was related to almost all aspects of human society. Min, who had majored in Chinese Language and Literature and had no comparable work experience before her practicum in Hong Kong SAR, China, believed in the inherent complexity of conceptualizing “culture” itself:

[1] If you want to define culture, um, it is a really broad term, including innumerable things. It may contain history, geography, human spirit, and so on. Oh, a lot of things, and it is an abstract concept! (Min)

Her view of culture tended to be static. Although Lan shared the same view that it was not clear what actually constituted culture, Lan regarded culture as a dynamic concept and said, “From tangible to intangible, culture is a huge system and comprises various dimensions.”

Despite their understanding that the concept of culture was ambiguous, most participants (6/7) reported that cultural knowledge in language education should be concrete and should include specific things, mainly Chinese historical stories, customs and habits, and geographical knowledge. For instance, Xiao, the only participant with 2 years of teaching experience at a secondary school in mainland China, talked about different ways of integrating culture into Chinese language classes and stated that a Chinese language teacher should be familiar with Chinese traditions and folk customs. Likewise, Min explicitly stated that “historical stories are mostly used” in her teaching. However, in Lan’s mind commonly used cultural words were also an essential part of a FL/SL learners’ linguistic repertoire in intercultural communication situations:

[2] I think, first of all, I need to teach the cultural words related to Chinese social contexts, such as some terms of respect, self-depreciatory expressions, and polite expressions. These are connected with the moral values in Chinese culture. (Lan)

The participants’ accounts suggested that they prioritized traditional cultural knowledge when teaching Chinese in the international school context. Specifically, they preferred to teach or transmit Chinese customs and habits, because they knew more about Chinese culture than about the cultures of other languages.

In addition, the majority of the participants were able to demonstrate their conceptual understanding of intercultural communication. They thought that issues concerning intercultural communication could arise for people with different cultural or language backgrounds, and even for people from different sub-cultures (Perry and Southwell, 2011). Xiao had obtained her Bachelor’s degree in Teaching Chinese as an International Language at a mainland Chinese university; she perceived that intercultural communicative competence was necessary when people experienced cultural differences:

[3] Language is the bridge of communication and is used to communicate among people. If two persons have different cultures, they may have intercultural issues when communicating with each other. This is true even for two persons from the same country if they come from different places and have different cultural norms. (Xiao)

Although the participants expected to benefit learners’ intercultural development through their teaching, most of them approached intercultural communicative competence from a cultural knowing viewpoint (Moran, 2001) and focused on the intercultural knowledge dimension.

### Chinese as an additional language teachers’ objectives in teaching intercultural communicative competence

This research also found that all the participants were consistently aware of the significance of developing students’ intercultural communicative competence in the Chinese language classroom. While different interviewees allocated different instruction time to intercultural teaching, they all held positive views of the need to improve students’ intercultural communicative competence. Intercultural knowledge refers to understanding “one’s own and other social groups’ cultural settings, norms, and interaction processes” (Gong et al., 2022, p. 134), and almost half of the participants (3/7) regarded this as an important objective in intercultural education, especially with regard to raising students’ interest in or motivation for Chinese language learning (Schmidtke-Bode and Kachel, 2020). For instance, Zeng advocated helping her students to concentrate on clear differences between their own and traditional Chinese cultures. She saw intercultural knowledge as a useful tool in the Chinese language classroom:

[4] In my mind, especially for foreign students, culture is a very good starting point in language education. This can help them like your class and can help them learn something different. Chinese traditional culture is very new to them because they do not have Chinese festivals or Chinese folk customs. They are interested in these things because they did not see them before. (Zeng)

In addition, all the participants’ responses (7/7) indicated that they believed that the aim in teaching intercultural communicative competence was not only to increase students’ intercultural knowledge, but also to improve their intercultural attitudes. For instance, Min took into account her students’ openness and tolerance toward other cultures, and she also encouraged them to learn to be more inclusive and open-minded:

[5] In my view, the ultimate aim of all cultural teaching is to help people with various cultural backgrounds interact more effectively. They can understand and be tolerant with each other, and differences in culture or values will cause no communication problems. (Min)

Similarly, Lu, the only male teacher among the seven participants, also perceived that students needed to improve their intercultural awareness with the ultimate goal of enhancing their intercultural communicative competence:

[6] You may find the (target) language they speak sounds awkward, but if they can learn some cultural content, they can use the target language more effectively and appropriately. In this process, they can also learn and absorb various cultures. This can help them acquire multicultural understanding and international mindedness. (Lu)

The participants’ accounts showed that “intercultural understanding” was frequently defined as the objective of intercultural education (e.g., Meng, Xiao). Moreover, Xiao favored promoting students’ critical thinking through her intercultural teaching, and argued:

[7] Through learning culture and intercultural content, students can broaden their horizons and will observe things more profoundly. This can improve their critical thinking, and at the same time, students can obtain the skills to learn other things and subjects. (Xiao)

Intercultural communicative competence considers the knowledge, attitudes, skills, and behaviors needed to take part in intercultural encounters. Byram (1997) identifies two factors of the skill dimension that affect intercultural communication, namely *savoir-comprendre* (skills of interpreting and relating) and *savoir-faire* (skills of discovery and interaction). The skills of interpreting and relating represent the balance between how people perceive their culture and how they can link local events with others from a different culture by interpreting and comparing (Fernández-Agüero and Chancay-Cedeño, 2019).

Our analysis indicated that three participants (3/7) concentrated on enhancing their students’ *savoir-comprendre* in Chinese language teaching. Specifically, they tended to use various cultural heritage forms to engage students in exploring diverse cultural manifestations. Ying provides a representative example of teaching intercultural skills:

[8] When I taught Chinese New Year culture, I showed them some videos or physical materials, like *Chunlian* (couplets). I wanted to provide multiple resources to construct a social scene, and let students be involved in it. If they (students) encounter some problems in communicating with Chinese people, teachers need to tell them about the cultural differences in this communication and compare different cultures. (Ying)

Moreover, Ying adopted an activity named “City DNA” to encourage students to “see a real scene outside the classroom.” Such strategic teaching efforts, shaped by the broad cultural diversity in her working community and Hong Kong society and mediated by her identity as an “intercultural learner” (Gong et al., 2022, p. 141), was in line with her tangible aim of intercultural teaching. Similarly, Lu expected that “students can tell the differences between their own cultures and Chinese culture” by adopting a cross-cultural comparison approach in the classroom. While the participants defined their teaching goals toward the full attainment of intercultural attitudes, knowledge, and skills, in fact the data recorded very few references to the dimension skills of discovery and interaction in Chinese education.

## Discussion

Teacher cognition concerning the general nature of intercultural communicative competence is considered an empirically relevant precondition for high quality intercultural education in language classrooms. There is a paucity of research examining preservice teacher cognition in developing students’ intercultural communicative competence in the context of teaching Chinese as an additional language. To fill this research gap, the present study has examined knowledge about and objectives in developing students’ intercultural communicative competence among a group of seven preservice teachers in a TCSL education program at Hong Kong SAR, China.

In terms of the participants’ knowledge about intercultural teaching, this research has revealed that all of them believed that intercultural communicative competence should be taught in Chinese language classes. This finding reflects the results of studies by Young and Sachdev (2011); Czura (2016), and Safa and Tofighi (2022), which all verified teachers’ positive inclinations toward a full attainment of intercultural communicative competence in EFL classrooms. In this regard, it seems that language teachers generally recognize the educational transition from a mainly linguistic focus to an intercultural development orientation (Tolosa et al., 2018; Gong et al., 2021).

Intercultural understanding and the development of student intercultural competence, which have recently been integrated into some national curricula and other educational documents, may positively shape and improve language teachers’ awareness to foster students’ intercultural communicative competence through their instructional efforts. In line with previous literature (Sercu, 2005; Gong et al., 2018a), our participants tended to define the concept of culture as static and ambiguous, mostly concentrating on the ways in which Chinese people live and their customs and traditions. Therefore, there seems to be no significant difference between preservice and in-service teachers concerning their beliefs about intercultural teaching (Safa and Tofighi, 2022).

According to the participants’ interview accounts, they benefitted considerably from the IB educational philosophy, their internship schools’ international environment, and Hong Kong SAR, China’s multilingual and multicultural context in terms of developing students’ intercultural communicative competence in their Chinese as an additional language classes. The preservice teachers with cross-border experiences showed a stronger awareness of intercultural teaching goals in their educational practices; in other words, it seems that preservice teachers in cross-border contexts can develop new and different insights into how to teach intercultural communicative competence and how to interpret their existing and future intercultural teaching practices (Sercu, 2006; Lai et al., 2016). Hence, there is a pressing need for educational policymakers to create intercultural and international settings or to provide intercultural communication opportunities for preservice teachers. Cross-border teacher education programs must continue to play an irreplaceable role in improving language teachers’ beliefs and practices in developing students’ intercultural communicative competence.

Regarding the participants’ objectives to develop their students’ intercultural communicative competence, this research indicates that they tended to relate the aim of intercultural teaching to attitudes more than to knowledge or skills. This finding was different from the results reported by Sercu (2005), Oranje and Smith (2018), and Ghavamnia (2020), who found that foreign language teachers primarily defined intercultural teaching in terms of cultural-knowledge transmission and did not aim to facilitate students’ development of intercultural attitudes or skills. It also differed from Gong et al.’s (2018a) investigation of Chinese as a second language teachers from universities in China, which found that the teachers rated promoting students’ intercultural skills to be the most important objective in their teaching, followed by the enhancement of students’ intercultural knowledge and promoting their ability to handle intercultural communications (the skill dimension). On the one hand, these inconsistent results show that the context of education and work may influence preservice and in-service teachers’ cognitions and practices in terms of intercultural teaching, since every teacher education or training program places a different degree of focus on intercultural teaching. Therefore, a comprehensive and consistent standard for preparing interculturally competent teachers is required, and it will be necessary to integrate intercultural development modules into SL/FL teachers’ education programs to qualify them to perform their future roles as teachers of both language and intercultural communicative competence (Larzen, 2005; Tolosa et al., 2018).

At the same time, since language teachers’ identity plays a crucial role in shaping their cognitions and practices in teaching intercultural communicative competence, an intercultural-identity-oriented approach also needs to be integrated into teacher education programs (Yang and Markauskaite, 2021). Further studies are also needed to indicate the interaction between preservice teachers’ identities and their intercultural teaching practices. Specifically, longitudinal investigations tracking changed in intercultural cognition and instructional practices during the transition from student teachers to experienced teachers may provide fresh insights for teacher education and development programs aiming to build language teachers’ intercultural identity (Nguyen and Yang, 2018).

## Conclusion

This exploratory qualitative study examined preservice Chinese language teachers’ cognitions related to developing their students’ intercultural communicative competence in the context of a TCFL education program in Hong Kong SAR, China. It expands our knowledge of how preservice teachers of non-Western languages perceive intercultural teaching in a cross-border setting. Overall, the findings of this study indicate that the participants held relatively positive attitudes and inclinations toward their students’ intercultural development, while understanding culture statically and ambiguously. Their intercultural pedagogical objective was more attitude-oriented than knowledge-oriented or skill-oriented. Their cross-border life, educational, and intern experiences may all have influenced their perceptions of the significance of facilitating students’ intercultural development in the Chinese as an additional language classroom.

The empirical evidence presented here might be different for preservice language teachers in different education systems, a recognition that calls for further studies on curricular innovations with intercultural aims, as well as the adoption of an intercultural approach in preservice teacher education programs and in-service teachers’ professional development courses. Also, in any qualitative inquiry researchers might impose their own beliefs about and interest in the discourses under study (Dervin, 2010; Gong et al., 2020). Our position as Chinese researchers means that to some extent our data interpretation might reflect our understanding of culture and intercultural communicative competence.

Despite these limitations, this study contributes new empirical evidence regarding preservice teachers’ knowledge about and their objectives in intercultural teaching in the language classroom. It has highlighted the crucial role of cognition in preservice teachers’ future instructional practices, and the support that seems to be required in order to assist their professional development. A longitudinal research design would be helpful in future research aiming to inform teacher professional development trajectories and tap into the changes in their belief systems and practices related to teaching intercultural communicative competence.

## Data availability statement

The raw data supporting the conclusions of this article will be made available by the authors, without undue reservation.

## Ethics statement

The studies involving human participants were reviewed and approved by the University of Macau. The patients/participants provided their written informed consent to participate in this study.

## Author contributions

YG designed the study, collected the data, analyzed the data, and wrote the manuscript. CL, XG, and GL reviewed the manuscript. YH collected the data. LL revised the manuscript. All authors contributed to the article and approved the submitted version.
